# Investigating the flexural performance of reinforced concrete beams using eco-friendly geotextile and wire mesh reinforcements

**DOI:** 10.1038/s41598-026-57299-3

**Published:** 2026-06-17

**Authors:** Bassant Hamed, Amr Aly Gamal El-Din, Sayed Salah, M. S. El-Feky

**Affiliations:** 1 Department of Structural Engineering, Faculty of Engineering, Benha University, Benha, Egypt; 2 Department of Civil Engineering, Modern Academy, Cairo, Egypt; 3 Department of Civil Engineering, National Research Centre, Cairo, Egypt; 4 Department of Civil Engineering, Heliopolis University, Cairo, Egypt

**Keywords:** Flexural performance, Strengthening, Geotextile, Wire mesh, Carbon nanotubes, Sustainability, Engineering, Materials science

## Abstract

This paper presents an experimental investigation on the flexural performance of reinforced concrete (RC) beams strengthened with eco-friendly materials, specifically steel wire mesh and geotextiles, in conjunction with carbon nanotubes (CNTs). Two strengthening techniques were investigated: (1) internal strengthening by embedding geotextile or steel wire mesh along the longitudinal reinforcement; (2) external strengthening by U-wrapping geotextile or steel wire mesh on the beam surface. Additionally, CNTs were incorporated into the concrete matrix as a third strengthening mechanism. Seven small-scale RC beams (600 × 150 × 150 mm) were tested under one-point bending. The findings demonstrate that all strengthened RC beams exhibited enhanced load-carrying capacity (up to 45% increase), ductility (up to 184% increase), and total energy absorption compared to un-strengthened specimens. Internal wire mesh (WF1) achieved the highest ultimate load (56.2 kN). External geotextile (PGF2) exhibited exceptional deformation capacity (32.7 mm deflection, 1376.9 kN·mm toughness). CNT-modified concrete demonstrated pseudo-ductile behavior with distributed micro-cracking and 666.3 kN·mm total toughness.

## Introduction

Reinforced concrete (RC) continues to serve as a fundamental component of contemporary infrastructure due to its versatility and strength. The composite action between concrete, which resists compression, and embedded steel reinforcement, which provides tensile capacity, has governed structural design for over a century. Nevertheless, the long-term sustainability of this traditional system is now under increasing scrutiny. The production of conventional steel reinforcement is a highly energy-intensive process, which is a significant contributor to global CO_2_ emissions and atmospheric pollution^1,2^. Moreover, the mining of iron ore and the processing of raw materials are associated with the degradation of habitats and the depletion of resources^3,4^. From a durability perspective, the inherent susceptibility of steel to corrosion in aggressive environments represents a critical limitation, often leading to cracking, spalling, and progressive structural deterioration^5,6^. The aforementioned degradation mechanisms necessitate frequent maintenance, repair, and premature replacement, thereby increasing material consumption, lifecycle costs, and overall environmental burden. Research Significance and Novelty: Despite extensive research on external strengthening using FRP composites and textile-reinforced mortars, several critical gaps remain unaddressed. First, no previous study has reported the use of woven geotextiles as internal reinforcement embedded within the concrete matrix—all existing applications are limited to external wrapping or soil stabilization. Second, a direct comparative evaluation of internal versus external placement of the same reinforcement material (woven geotextile or wire mesh) is lacking in the literature. Third, the combined effect of CNT matrix modification with discrete geotextile or wire mesh reinforcement has not been systematically investigated. The present study addresses these gaps by: (i) evaluating woven geotextiles as both internal and external flexural reinforcement; (ii) directly comparing internal vs. external strengthening performance for two sustainable materials; and (iii) assessing the synergistic potential of CNTs to enhance matrix toughness in combination with macro-reinforcement. Consequently, the significant environmental and economic costs associated with conventional steel reinforcement are driving intensive research into alternative, more sustainable materials that can complement or partially replace steel^7,8^.

To enhance the flexural performance of RC beams, numerous strengthening materials and techniques have been proposed and extensively investigated in the literature^9^. These include externally bonded fiber-reinforced polymer (FRP) composites, such as carbon (CFRP), glass (GFRP), and basalt (BFRP) systems, which have demonstrated high strength-to-weight ratios and effective improvements in flexural capacity^10–13^. In addition, textile-reinforced mortar (TRM) and fabric-reinforced cementitious matrix (FRCM) systems have gained increasing attention due to their improved fire resistance, vapor permeability, and compatibility with concrete substrates^14,15^. Beyond these conventional approaches, increasing interest has been directed toward alternative textile- and geosynthetic-based reinforcements, such as geotextiles and geogrids, as well as distributed metallic systems including welded wire mesh, owing to their cost-effectiveness, corrosion resistance, and ability to provide distributed tensile reinforcement and crack control. Moreover, recent advances in nanotechnology have enabled the modification of cementitious matrices using nanomaterials, particularly carbon nanotubes, which have shown strong potential for enhancing matrix strength, refining pore structure, and promoting microcrack bridging at the nano-scale^16–20^.

Building upon the principles of externally bonded reinforcement, high-strength geotextile composites present a sustainable alternative to conventional FRP systems for flexural strengthening. These polymer-based materials offer several environmental advantages, including potentially lower embodied energy in production and reduced carbon emissions compared to carbon fiber systems. When applied as externally bonded reinforcement to the tension face of RC beams, geotextile composites provide additional tensile resistance that directly enhances flexural capacity^18–22^. while a review of the literature indicates that, to date, no studies have reported their use as internal reinforcement within the concrete matrix, highlighting a clear research gap, and motivating this study to assess their effectiveness in enhancing ultimate moment capacity, controlling crack widths, and improving ductility. Recent studies have explored alternative sustainable reinforcements^10,19,21,22^.

Welded wire mesh (WWM) is widely used as externally bonded reinforcement for the flexural strengthening of reinforced concrete beams, providing enhanced tensile resistance, crack control, and deformation performance while promoting durable and resource-efficient solutions^18,23,24^; however, to date, only a single study has explored its use as internal flexural reinforcement^25^. Similarly, the incorporation of carbon nanotubes (CNTs) transforms the concrete matrix into a more resilient composite material, reducing the need for conventional steel reinforcement through intrinsic material enhancement^19,26^. CNTs enhance tensile performance by bridging microcracks and improving the interfacial transition zone^27,28^. Yet, no study has systematically compared internal versus external placement of WWM or woven geotextiles, nor investigated CNTs combined with these discrete reinforcements. This study fills those gaps.

In response, research is increasingly focusing on innovative, eco-friendly materials specifically designed for the flexural strengthening and retrofit of RC members. Beyond merely substituting conventional steel, these materials offer additional functionalities, including enhanced crack control, improved strength-to-weight ratios, and corrosion resistance. In the present study, high-strength geotextiles and welded wire mesh (WWM) were applied as externally or internally bonded reinforcement, while carbon nanotubes (CNTs) were incorporated independently into the concrete matrix to enhance its intrinsic flexural performance. Each material was examined according to its distinct contribution: geotextiles and WWM for supplemental reinforcement, and CNT-modified concrete for intrinsic improvement of tensile behavior.

## Experimental program

### Materials

**Fig. 1:**
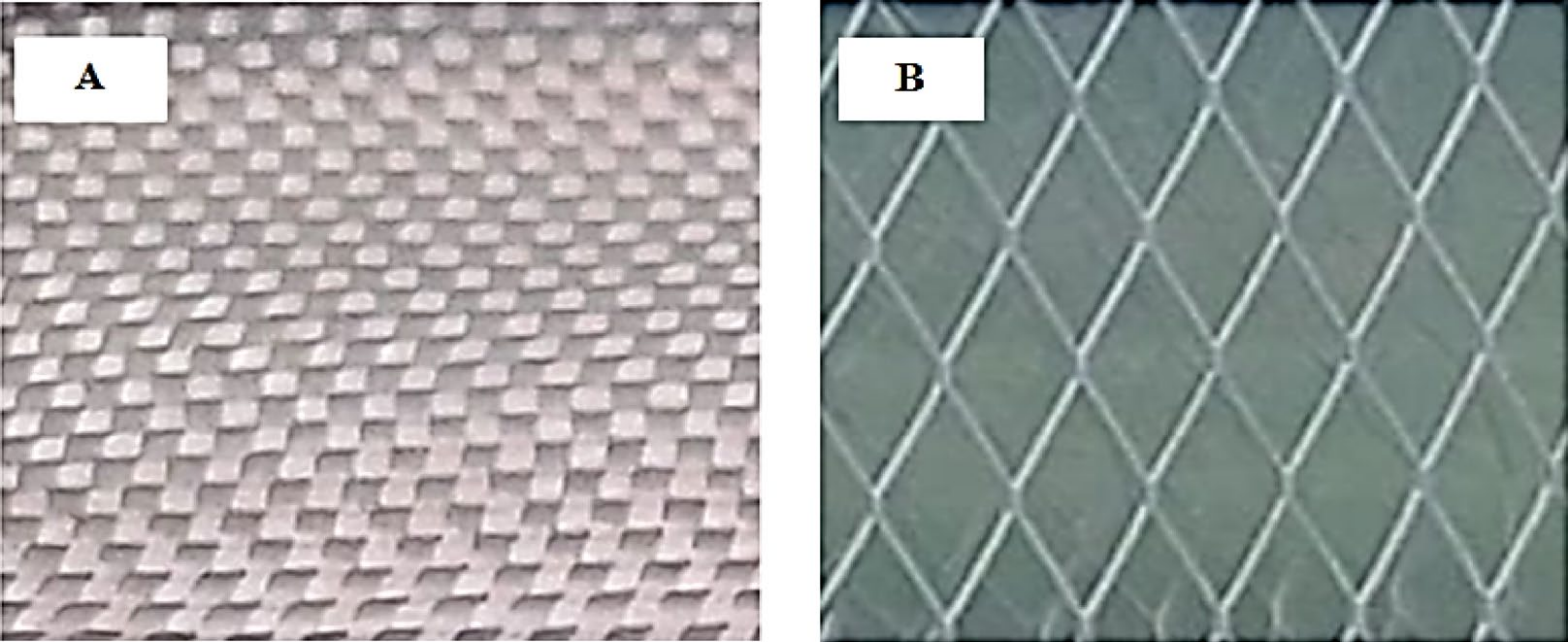
The used strengthen materials of RC beams; (**A**) Plain geotextile (PG) and (**B**) steel wire mesh (SWM).

Internal and external strengthening of the RC beams was achieved using two types of materials, plain geotextile (PG) and steel wire mesh (SWM) as shown in Fig. 1, whereas carbon nanotubes (CNTs) were used as an additive to enhance the concrete mix.

Woven Geotextile (PG) – The reinforcement used is a woven polypropylene geotextile. The geotextile has a plain weave pattern with warp and weft yarns of 1000 tex each. The following properties were determined according to ASTM D4595 (tensile)^29^ and ASTM D5199 (thickness)^30^:

- Thickness: 0.6 mm.
- Mass per unit area: 320 g/m^2^.
- Tensile strength (warp direction): 45 kN/m.
- Tensile strength (weft direction): 40 kN/m.
- Elongation at break (warp): 12%.
- Elongation at break (weft): 14%.
- Elastic modulus (secant at 2% strain): 5.2 GPa.
- Bond strength with concrete (pull‑out test, 150 mm embedded length): 0.9 MPa.
- Aperture size: 1.5 mm × 1.5 mm.

Steel Wire Mesh (SWM) Specifications: The steel wire mesh employed in this study is a galvanized welded steel wire mesh (BRC 50 × 50) used in this study had a grid spacing of 50 × 50 mm and a wire diameter of 2.0 mm. The material properties, obtained from the manufacturer and verified via coupon tensile testing per ASTM E8/E8M^31^, are summarized in Table 1.

**Table 1:** Physical and mechanical properties of the galvanized welded steel wire mesh.

Property	Value
Mesh type	BRC 50 × 50 (galvanized welded)
Grid spacing (mm)	50 × 50
Wire diameter (mm)	2.0
Sheet dimensions (m)	2.4 × 1.2
Young’s modulus (GPa)	200
Poisson’s ratio	0.30
Density (kg/m^3^)	7850
Yield stress (MPa)	210
Ultimate tensile strength (MPa)	350
Elongation at break (%)	12

#### Carbon nanotube dispersion protocol

The multi-walled carbon nanotubes (MWCNTs) used in this study have the following characteristics: inner diameter = 5 nm, outer diameter = 8 nm, length = 1–2 μm, purity > 95%, specific surface area = 250–300 m²/g. To ensure uniform dispersion within the cementitious matrix, a two-step dispersion protocol was employed:

Step 1 - Ultrasonic Dispersion: MWCNTs were added to deionized water at a concentration of 1 g/L. A polycarboxylate-based superplasticizer (SP) was added at a 1:1 weight ratio relative to CNTs as a surfactant. The suspension was subjected to ultrasonic agitation using a probe sonicator for 60 min in an ice bath. Ultrasonic energy was applied in 10-minute pulses with 2-minute cooling intervals.

Step 2 - Integration into Concrete Mix: The CNT suspension was mixed with dry cement for 5 min at low speed (140 rpm). Subsequently, aggregates and remaining water were added, and mixing continued for 10 min at high speed (285 rpm). The total CNT content was 0.045 kg per m³ of concrete (0.01% by weight of cement).

**Fig. 2:**
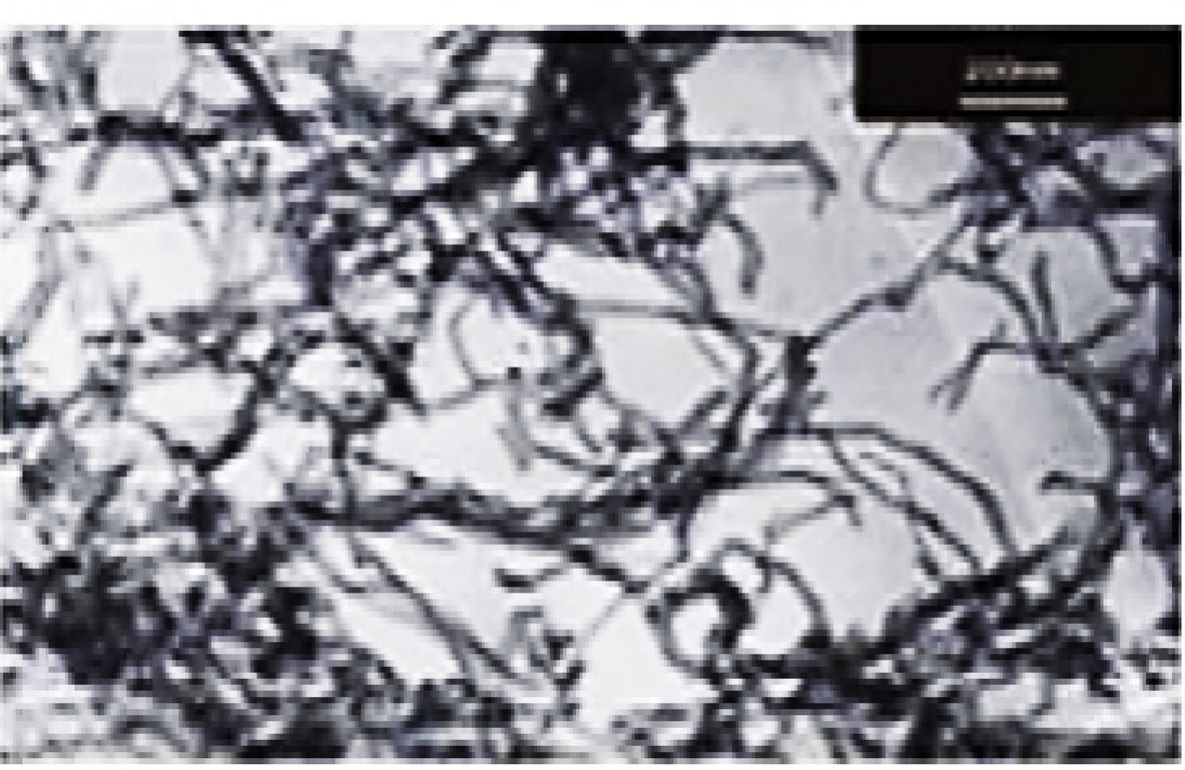
The SEM of carbon nanotubes.

A scanning electron microscope (SEM) image of the carbon nanotubes is presented in Fig. 2.

### Mixing sequence and preparation of the specimens

Two concrete mixes are prepared in this study, R mix is made of cement (C), coarse aggregates (CA), fine aggregate (F.A) water (W) and superplastizer (SP), while RCNT mix is made in the same manner but with adding carbon nanotubes (CNTs) as an additive to cement content. The water cement ratio is set to a steady value of 0.43 for all mixes. The aggregates for all mixes formed of crushed dolomite and fine sand with percentage of 65% and 35% by weight of total aggregates, respectively. The components of concrete mixes are represented in Table 2. After the concrete mixing process was completed, the fresh concrete was poured directly into well-cleaned and oiled steel molds in three layers. Each layer was compacted using a vibrating table, after which the surface of each beam was leveled, all beams were de-molded after 24 h and subsequently cured in fresh water at room temperature until the day of testing. Additionally, 100 × 100 × 100 mm³ concrete cubes were prepared and cast for compressive strength testing after 28 days of water curing at room temperature in accordance with BS EN 12390-3^32^. All specimens were cured in a temperature-controlled water tank maintained at 23 ± 2 °C for 28 days; water temperature was monitored daily and kept constant using a thermostatic controller.

**Table 2:** The components of concrete mixes (kg) per 1 m^3^.

Mix	C	C.A	F.A	W	w/c ratio	SP	(CNTs)	
							kg	%
R	450	1168	629	180	0.4	3.3	–	–
RCNT	450	1168	629	180	0.4	3.3	0.045	0.01%

### Details of tested beams

**Fig. 3:**
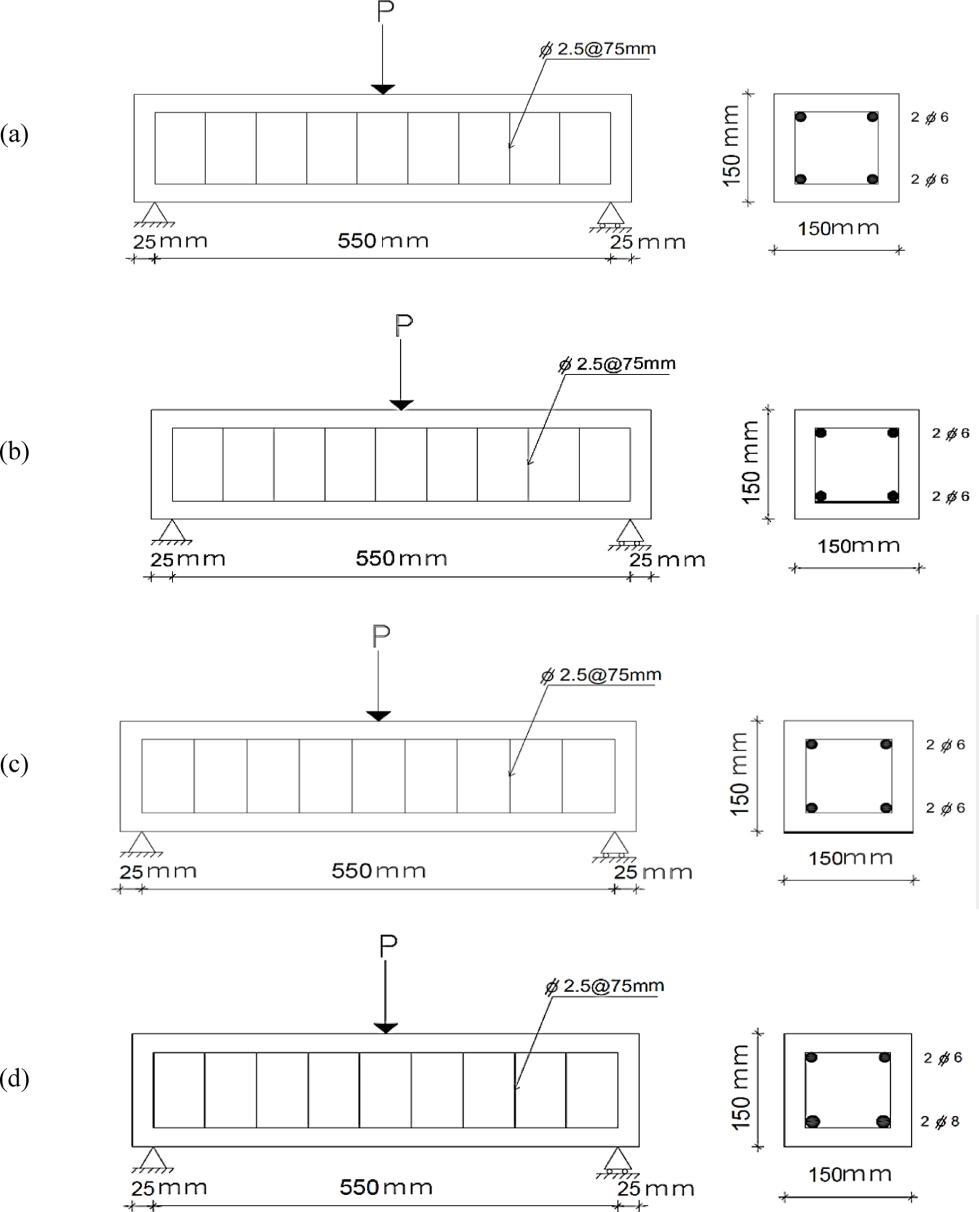
Beam specifications and corresponding strengthening schemes; (**a**) Flexural reference beam (RB-F) and Strengthened concrete mix(CNT-F); (**b**) Internal Strengthened RC beams (PGF1 and WF1); (**c**) External Strengthened RC beams (PGF2 and WF2); (**d**) reference beam (RB).

In this study, an overall of seven different RC beams were prepared, at small-scale specimen with dimensions of 600 mm in length, 150 mm in depth, and 150 mm in width. these beams were categorized into four different groups based on used strengthening technique; unstrengthen (reference beams), internal strengthening by either plain geotextile or steel wire mesh, external strengthen by either plain geotextile or steel wire mesh, and finally by strengthening the concrete mix with using carbon nanotubes (CNTs) as shown in Table 3. Beam specifications and corresponding strengthening schemes were shown in Fig. 3.

In specimens incorporating internal strengthening, a 100 mm‑wide strip of either woven geotextile or steel wire mesh was placed in a U‑shaped configuration around the longitudinal tension reinforcement bars only within the mid‑span region (central 300 mm of the 550 mm span). The reinforcement was secured using epoxy adhesive at 100 mm intervals. The U‑wrap extended along the bottom face (full 150 mm width) and up both sides (50 mm height on each side).

**Fig. 4:**
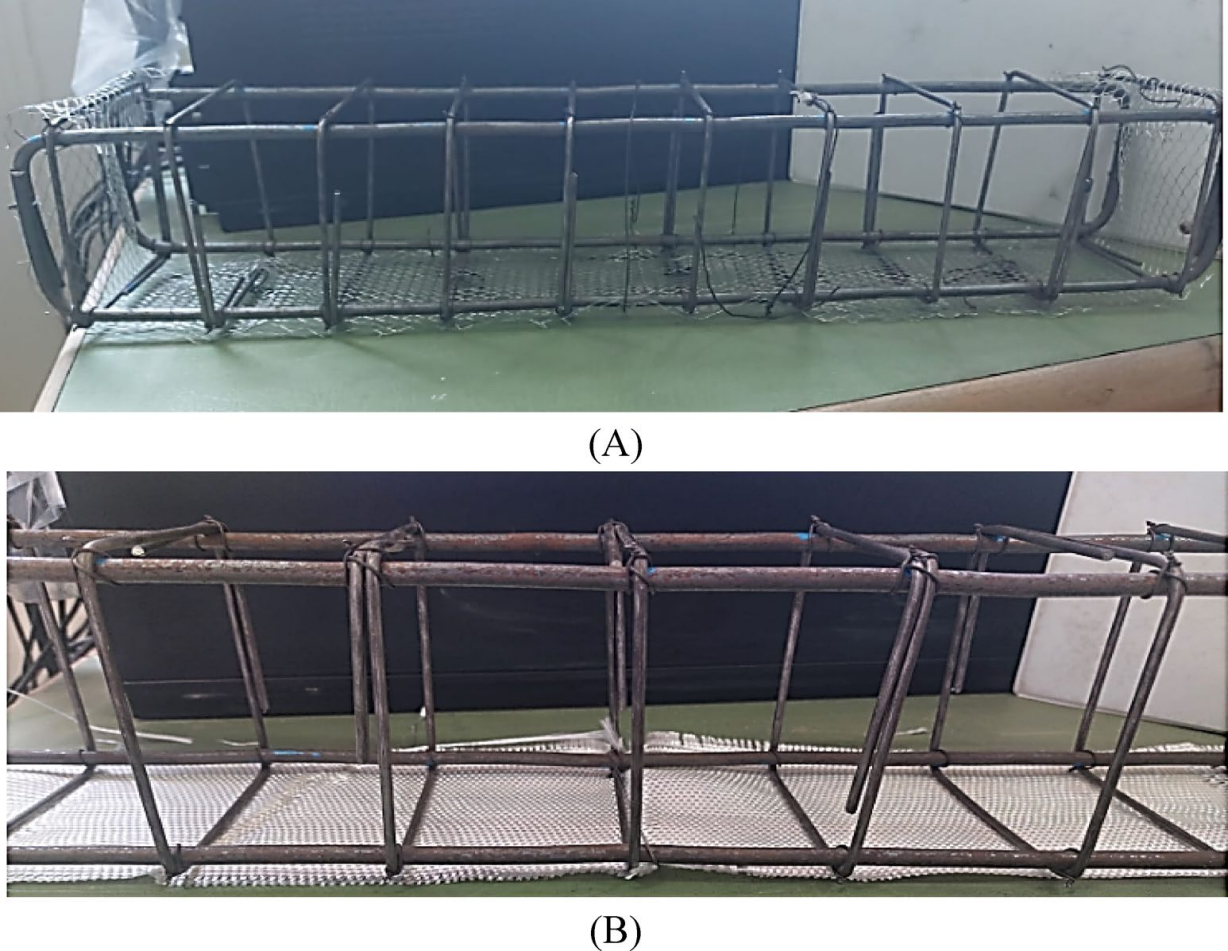
The reinforcement details for internally strengthened RC beams; (**A**) WF1 beam and (**B**) PGF1 beam.

**Fig. 5:**
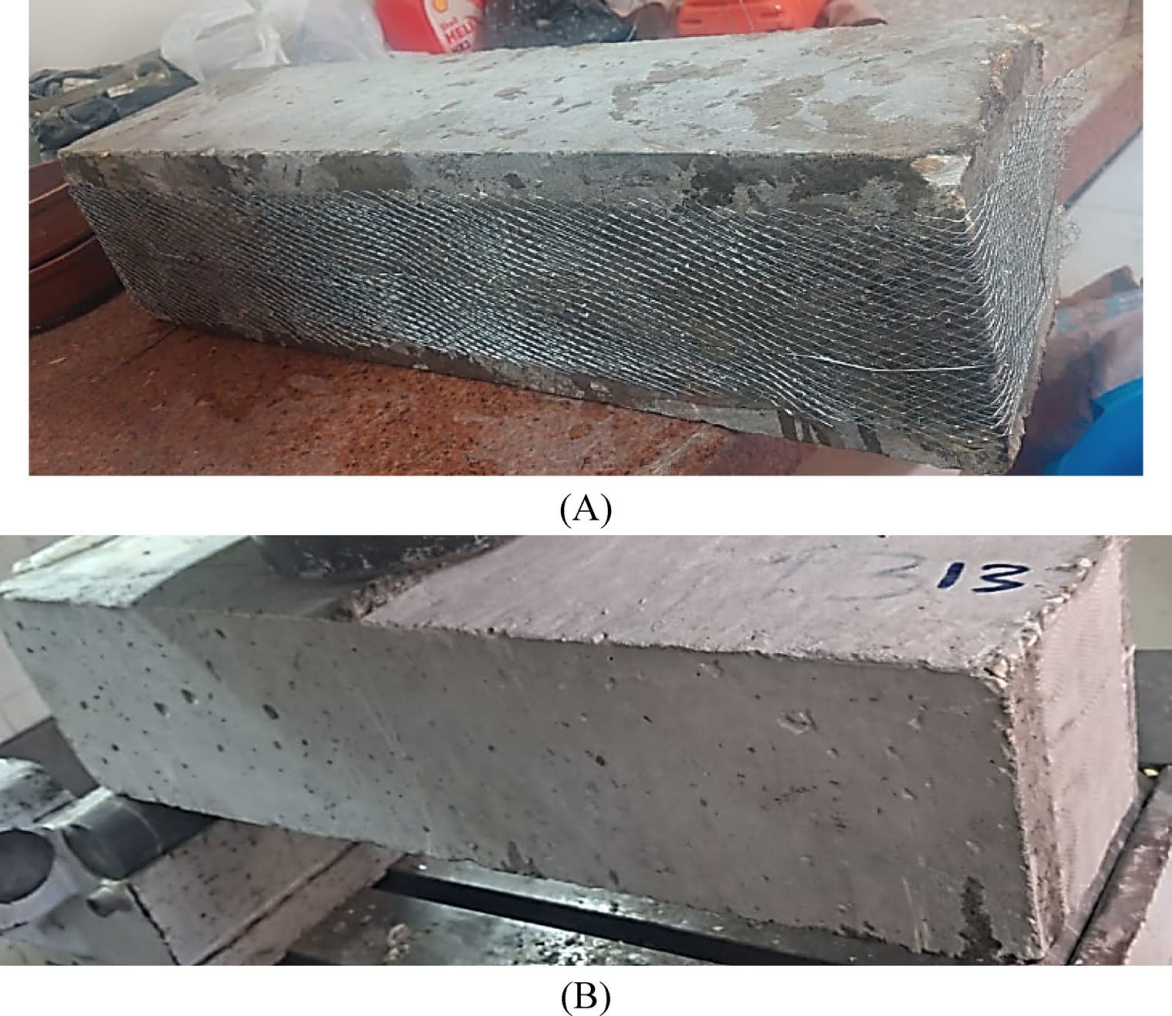
The reinforcement details for externally strengthened RC beams; (**A**) WF2 beam and (**B**) PGF2 beam.

For externally strengthened specimens, the same 100 mm‑wide strips were applied as U‑shaped external wraps on the beam surface, covering the same mid‑span region. Surface preparation included sandblasting and cleaning with compressed air before epoxy bonding. As illustrated in Figs. 4, and 5.

**Table 3:** The strengthened RC beams for flexural performance.

Strengthening technique	Specimen ID	Strengthening material	Strengthening shape
Unstrengthen RC beam	Reference beam (RB)	Nil	Nil
	Flexural reference beam (RB-F)	Nil	Nil
Internal strengthened RC beam	PGF1	Geotextile	One strip of 100 mm width is fully U- wrapping
	WF1	Steel wire mesh	
External strengthened RC beam	PGF2	Geotextile	
	WF2	Steel wire mesh	
Strengthened concrete mix	CNT-F	Carbon nanotubes	Dispersed in concrete mix

### Laboratory test set-up

**Fig. 6:**
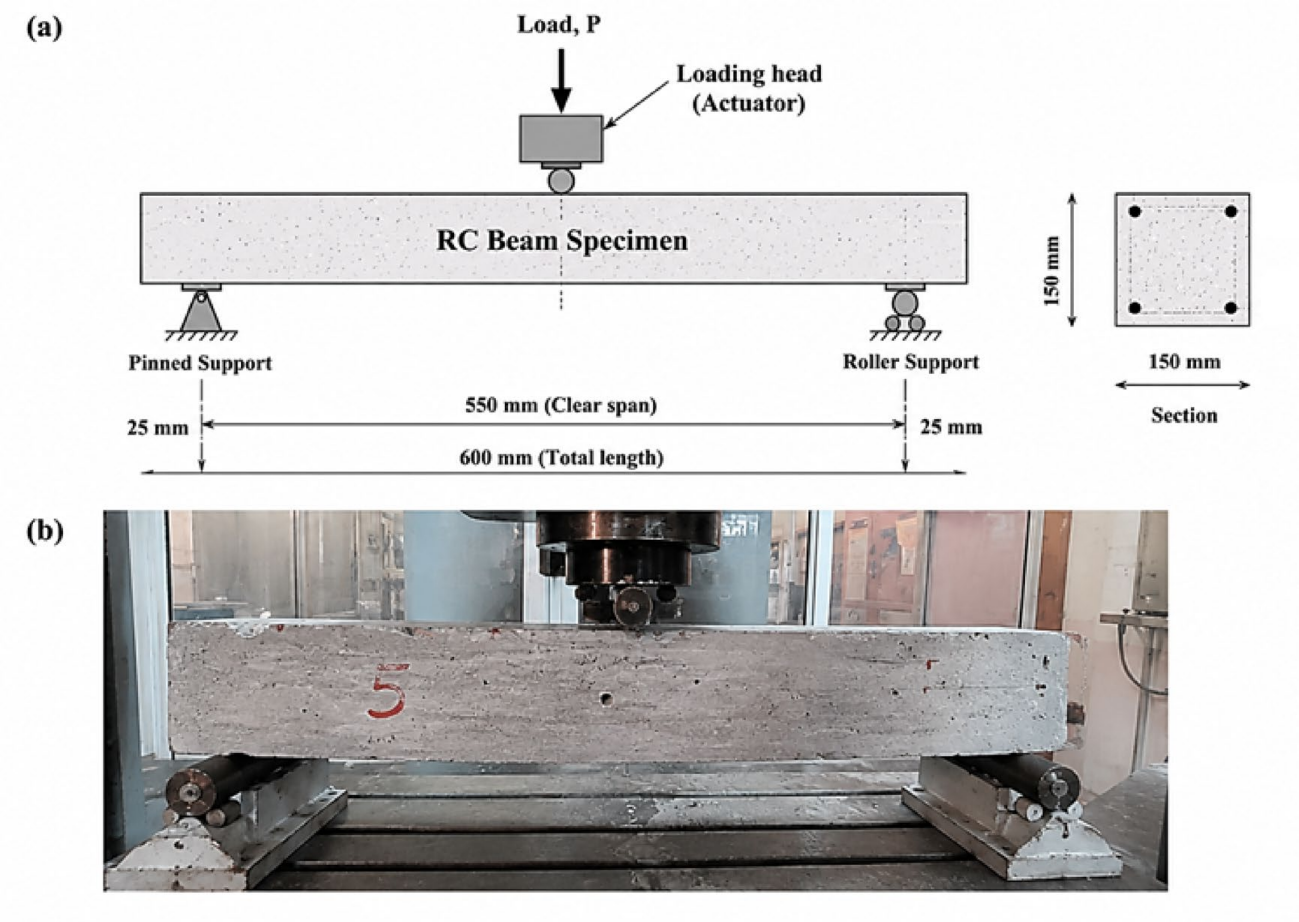
Flexural strength test setup using the SHIMADZU 1000 kN universal testing machine: (**a**) schematic illustration of the test setup; and (**b**) photograph of the experimental setup.

a one-point bending loading test setup was used. All RC beam specimens, both strengthened and un-strengthened, were tested at a constant rate of 0.05 mm/s using Universal testing machine SHIMADZU. Each beam was simply supported over a span of 550 mm, and the load was applied at mid-span through a hydraulic loading system. Load and deflection measurements were recorded continuously to capture the structural response of the specimens. The setup ensured accurate application of the load and proper alignment of the beams throughout the test, as illustrated in Fig. 6.

### Toughness (energy absorption) calculation

Toughness was calculated as the area under the load‑deflection curve using the trapezoidal integration method (MATLAB R2023a). Three parameters were defined:

- Pre‑yield toughness (Ty): area from origin to yield point (Py, Δy).
- Post‑yield toughness (Tp): area from yield to ultimate failure (Pu, Δu).
- Total toughness (Tt): Ty + Tp.

The yield point was determined using the 0.2% offset method (ASTM E8/E8M^31^ for specimens with distinct yielding, and the maximum curvature method for others (e.g., CNT‑F).

## Results and discussion

### Load- deflection behavior

**Fig. 7:**
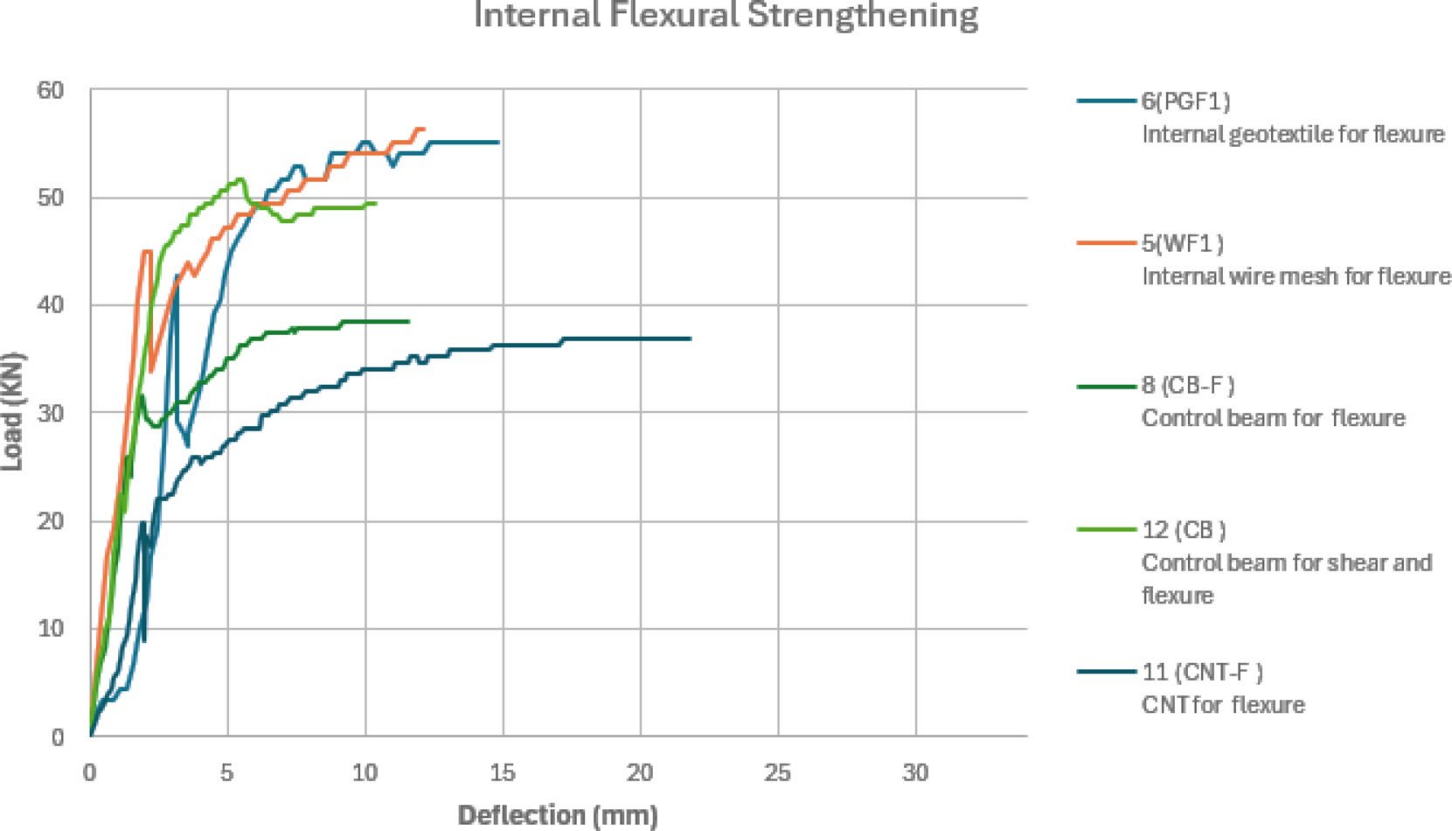
Load-deflection curves for internally strengthened beams.

**Fig. 8:**
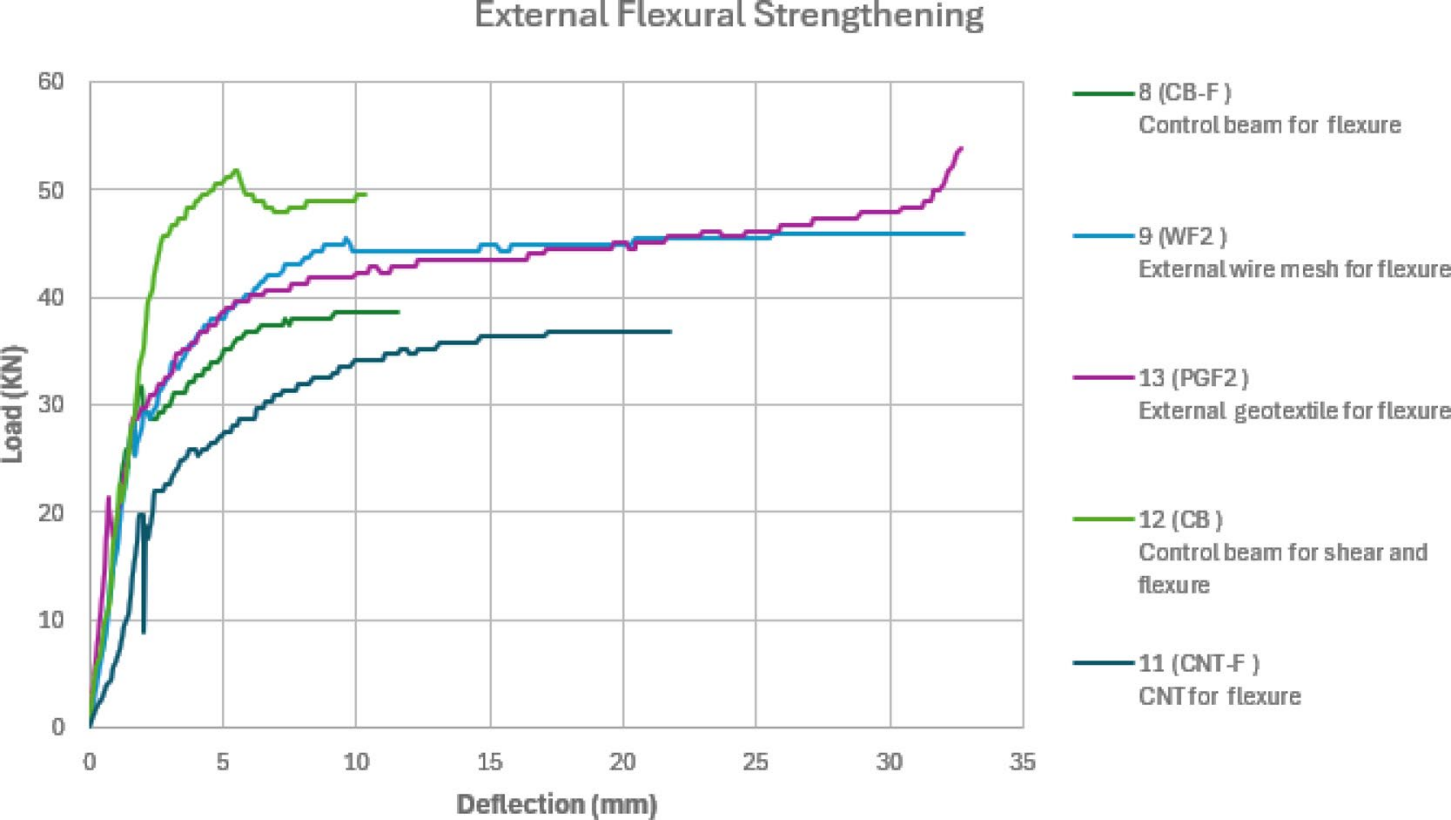
Load-deflection curves for externally strengthened beams.

The primary metrics here are the maximum load carried and the deflection at failure, which indicate strength and ductility, respectively. Figures 7 and 8 show the load- deflection curves for internal and external strengthen beams respectively and Table 4 illustrates the summary of test results.

For control beam (CB-F), this beam serves as the control. It shows a typical quasi-brittle failure pattern. The load increases until a peak of approximately 38.5 kN at a deflection of about 11.5 mm, after which it fails suddenly.

For Internally Reinforced Beams (PGF1 & WF1), Internal Geotextile (PGF1) Shows a significant improvement over the control. It reaches a peak load of ~ 55.13 kN and exhibits a remarkably long, ductile plateau, deflecting over 14 mm while maintaining most of its load. This indicates that the geotextile effectively bridges cracks after the concrete cracks, allowing for large, stable deformations without collapse. The curve shows a “plastic” behavior, which is highly desirable for structural safety. While Internal Wire Mesh (WF1) is the strongest specimen in flexure. It achieves a remarkable peak load of ~ 56.25 kN and also demonstrates extreme ductility, deflecting beyond 11 mm at a near-constant load. The wire mesh provides superior tensile strength, resulting in both high strength and high ductility.

For Externally Reinforced Beams (PGF2 & WF2), External Geotextile (PGF2) Performs well, reaching a final load of about 53.9 kN with very high ductility (deflecting over 30 mm). Its load-deflection curve shows a gradual strength increase, which is a desirable failure mode, and External Wire Mesh (WF2) is also an excellent performer. It reaches a similar final load as PGF2 (~ 46 kN) but shows a stiffer initial response and maintains its load over an even larger deflection (beyond 30 mm), indicating outstanding post-yield behavior.

For CNT Reinforced Beam (CNT-F), The CNT-F beam exhibits a unique and highly favorable response. It does not have a sharp peak. Instead, its load increases steadily to around 36–37 kN and then enters a very long, slightly hardening/plateauing phase, deflecting all the way to ~ 21.8 mm. This suggests that the CNTs act at a micro-level, effectively distributing stresses and preventing the localization of cracks, leading to a pseudo-ductile failure even in a typically brittle material.

For the Control Specimen (CB): indicate a peak load capacity of 51.7 kN, followed by a sudden loss of structural integrity. Once the ultimate load was attained, the specimen underwent a rapid strength degradation, with the load falling abruptly to 49 kN. This response is indicative of an unstable post-peak behavior, where the specimen failed to exhibit any substantial ductile deformation.

**Table 4:** The summary of test results.

Specimens	Yield stage		Ultimate load	Ultimate deflection
	P_y_(KN)	$\:{\varDelta\:}_{y}$(mm)	P_u_ (KN)	$\:{\varDelta\:}_{u}$(mm)
CB	46.2	2.99	51.7	10.3
CB-F	31.6	1.9	38.5	11.5
PGF1	42.7	3.15	55.1	14.8
WF1	45	2	56.2	11.9
PGF2	28.6	1.63	53.9	32.7
WF2	29.3	2	46	32.6
CNT-F	19.8	1.9	36.8	21.8

### Quantitative ductility and stiffness analysis

The ductility index (µ = Δu/Δy) and stiffness parameters were calculated for all specimens (Table 5).

**Table 5:** Ductility and stiffness parameters.

Specimen	Δy (mm)	Δu (mm)	µ	Ki (kN/mm)	Ky (kN/mm)	Ky/Ki
RB-F	1.90	11.5	6.05	21.3	16.6	0.78
PGF1	3.15	14.8	4.70	18.5	13.6	0.74
WF1	2.00	11.9	5.95	27.5	22.5	0.82
PGF2	1.63	32.7	20.06	21.9	17.5	0.80
WF2	2.00	32.6	16.30	18.4	14.7	0.80
CNT-F	1.90	21.8	11.47	16.1	10.4	0.65
CB	2.99	10.3	3.44	21.5	15.4	0.72

Externally strengthened specimens exhibit exceptional ductility (µ up to 20.1). WF1 shows the highest initial stiffness (27.5 kN/mm). CNT‑F has the lowest stiffness degradation (0.65), confirming distributed micro‑cracking.

### Scientific analysis of crack patterns in reinforced concrete beams

**Fig. 9:**
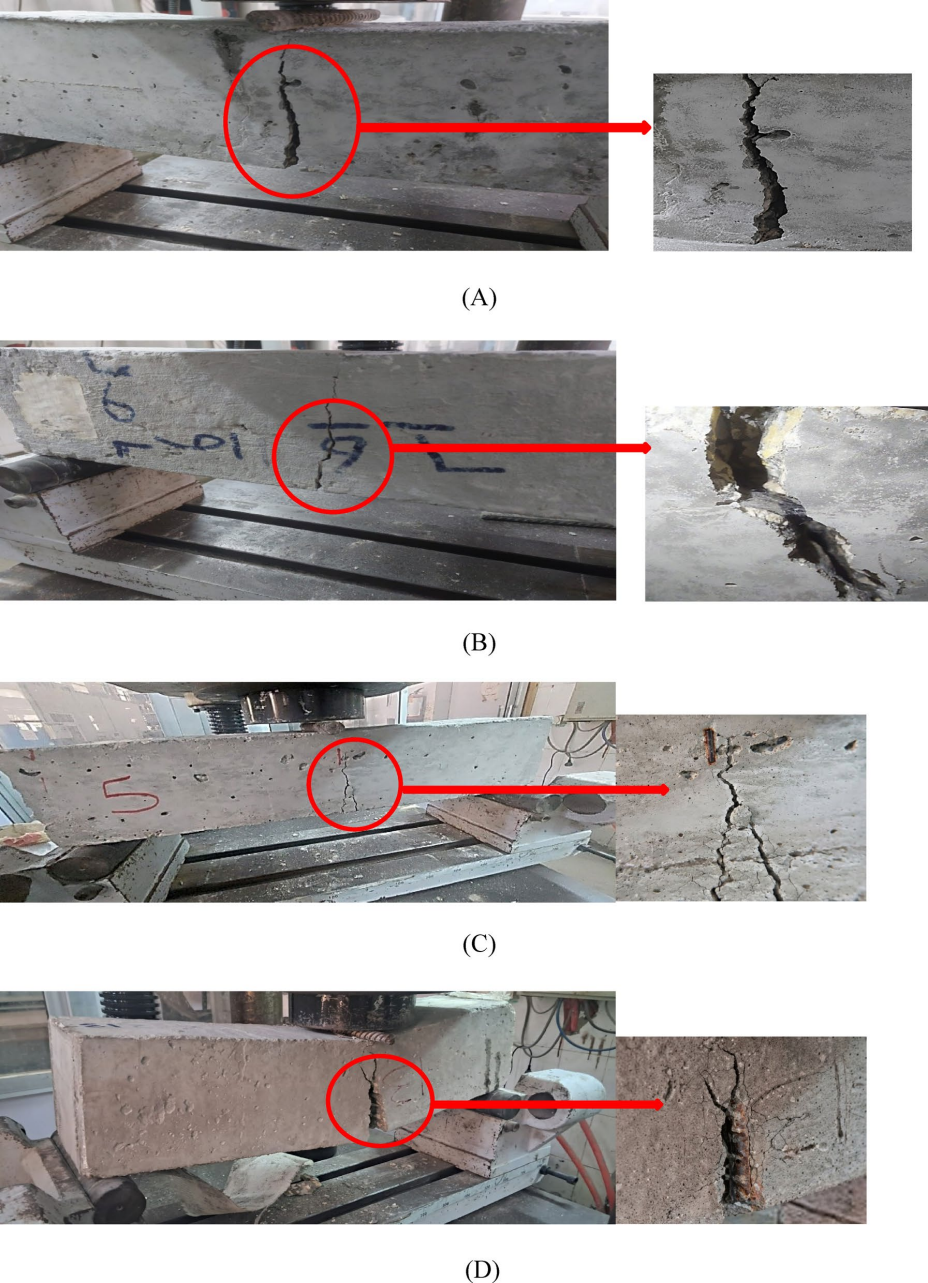

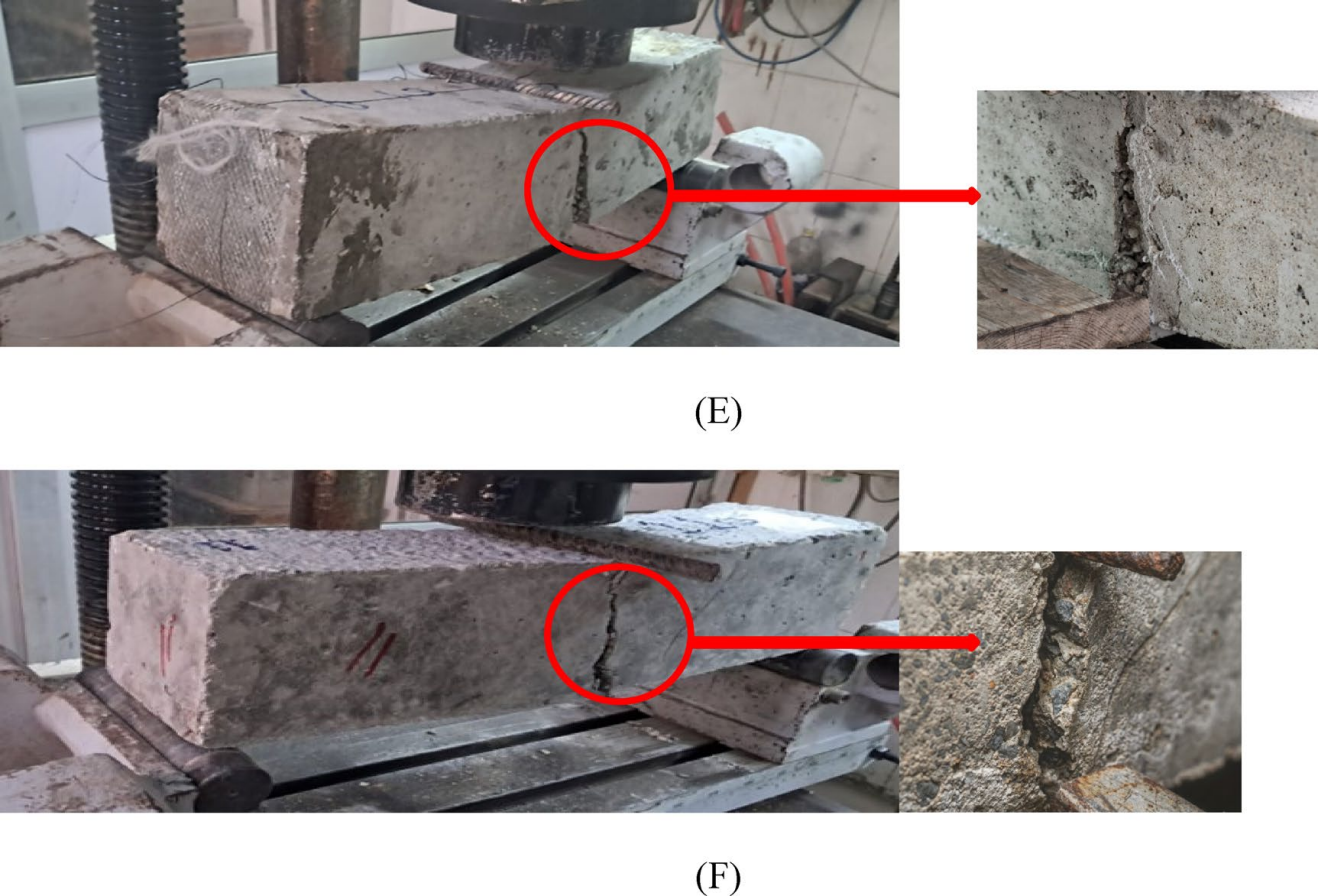
Crack patterns and failure modes: (**A**) RB‑F (control) – single brittle crack; (**B**) PGF1 – bridged macro‑crack with woven geotextile yarn pull‑out; (**C**) WF1 – multiple fine distributed cracks; (**D**) PGF2 – debonding progression with substrate cracks; (**E**) WF2 – debonding and mesh rupture; (**F**) CNT‑F – diffuse micro‑cracking zone (whitened area).

Based on the experimental data provided and the critical observation that all significant cracks are localized at the mid-span region, the following is a complete scientific discussion. This analysis discusses the findings for a dominant flexure interaction failure mode at the beam’s most stressed section. The failure mode and crack pattern of a reinforced concrete beam are direct visual manifestations of its internal stress distribution, material properties, and reinforcement effectiveness. The observed behaviors from the load-deflection curves are intrinsically linked to how cracks initiated, propagated, and were controlled. Figure 9 shows the crack pattern and mode of failure for the tested beams.

For control beam (CB-F), a Classic Brittle Flexural Crack was exhibited. A single, vertical-to-slightly-inclined tensile crack initiated at the bottom fiber at the point of maximum bending moment (mid-span). Upon reaching the concrete’s tensile strength (~ 38.5 kN), this crack propagated unstably upward through the depth, leading to immediate rupture of the concrete matrix. The crack faces were clean with minimal secondary micro-cracking, indicating no stress redistribution. The sharp peak and catastrophic load drop in the load-deflection curve are the direct graphical representation of this unstable crack propagation. The low energy absorption (small area under the curve) correlates with the minimal surface area created by a single failure plane.

For Internally Reinforced Beams (PGF1 & WF1), PGF1 (Internal Geotextile) displayed a Multiple Cracking Ductile Pattern. After initial micro-cracking, several discrete, vertically aligned flexural cracks formed within the constant moment region. The geotextile’s high strain capacity and bond strength allowed these cracks to open progressively. The final failure was characterized by one crack widening significantly into a bridged macro-crack, with the geotextile fibers visibly elongating and de-bonding over a length (“pull-out” mechanism), rather than rupturing. While WF1 (Internal Wire Mesh) showed a Finely Distributed Multiple Cracking Pattern. The high stiffness and tensile strength of the wire mesh led to the formation of numerous, closely spaced, hairline flexural cracks. The mesh effectively restrained crack widening, leading to a near-perfect plastic hinge formation. Failure likely involved yielding of the mesh, followed by concrete crushing in the compression zone or rupture of the mesh at ultimate strain. The flat plateaus in their load-deflection curves are the macroscopic result of sequential crack formation and stabilization. Each new crack or widening of an existing crack corresponds to a minor load redistribution, creating “plastic” behavior. WF1’s higher peak load (~ 56.2 kN vs. ~55.1 kN) reflects the wire mesh’s higher tensile strength and modulus compared to the geotextile.

For Externally Reinforced Beams for Flexure (PGF2 & WF2), Crack Pattern for both two beams were demonstrated a Substrate Cracking with Debonding Progression. Multiple flexural cracks first formed in the concrete substrate. The externally bonded reinforcement (EBR) then bridged these cracks. The primary failure mechanism shifted from material rupture to interfacial debonding. Crack patterns would show substrate cracks aligned with the eventual peeling or end-debonding of the fabric/mesh. The final failure is a composite one: concrete cracking + bond failure. The exceptional ultimate deflection (> 30 mm) is enabled by the large deformation capacity of the EBR system before debonding completes. The gradual post-peak hardening in GF2’s curve suggests progressive debonding, whereas WF2’s stiffer plateau indicates better bond integrity until a more sudden failure.

For CNT Reinforced Beam for Flexure (CNF-F), Diffuse Micro-Cracking with No Localized Macro-Crack is exhibited. The CNTs, acting as nano-scale reinforcement, significantly improve the fracture toughness of the cement matrix. This inhibits the initiation and coalescence of micro-cracks into a dominant crack. Instead, a wide band of distributed micro-damage forms at mid-span, often visible as a whitened, opaque zone due to the multitude of micro-cracks. The unique “deflection-hardening” or extended plateau response (to ~ 21.8 mm) is the direct consequence of this distributed damage. Energy is absorbed through the creation of vast micro-crack surface area rather than through a single crack. The absence of a sharp peak signifies the lack of unstable crack propagation.

### Energy absorption (toughness) results

Toughness, defined as the total energy absorbed prior to failure and represented by the area under the load-deflection curve, provides a critical metric for evaluating structural resilience. The calculated components—pre-yield toughness (Ty, elastic energy), post-yield toughness (Tp, plastic deformation energy) and total toughness (Tt).

The toughness data for flexural specimens strongly supports behavioral rankings and crack pattern analyses.

Control Beam (CB-F): Exhibits the lowest total toughness (Tt = 376.86 kN.mm). Its low post-yield toughness (Tp = 344.8 kN.mm) quantitatively confirms the brittle failure described, characterized by a single, unstable crack propagation offering minimal energy dissipation after the elastic limit.

Internally Reinforced Beams (PGF1 & WF1): Both show a significant increase in total toughness compared to CB-F, but through different mechanisms, aligning with their distinct crack patterns.

WF1 (Tt = 544.2 kN.mm): Possesses the highest pre-yield toughness (Ty = 45.6 kN.mm) among flexural specimens, correlating with its noted high stiffness and superior tensile strength. This high Ty reflects the energy absorbed in forming its distributed, fine micro-crack network before yield. Its substantial Tp (498.6 kN.mm) corresponds to the stable, ductile plateau resulting from wire mesh yielding and concrete crushing.

PGF1 (Tt = 622.4 kN.mm): Demonstrates a lower Ty than WF1 but a significantly higher Tp (583.7 kN.mm). This data directly supports the observation of its “exceptional, stable ductility.” The high Tp quantifies the immense energy dissipated through the wide, bridged macro-crack and the extensive fabric pull-out mechanism, creating the pronounced plastic plateau.

Externally Reinforced Beams (PGF2 & WF2): These specimens show an extraordinary increase in total toughness (Tt ~ 1376 kN.mm), the highest in flexural tests. This is almost exclusively due to their massive post-yield toughness (Tp > 1340 kN.mm), which is 3–4 times greater than their internal counterparts. This quantitatively validates the described “exceptional ductility” and deflection capacity exceeding 30 mm. The high Tp results from the extensive debonding process of the external reinforcement, which allows for very large deflections while maintaining a significant residual load, as shown in their load-deflection plateaus.

CNT Reinforced Beam (CNT-F): Presents a unique toughness profile: the lowest pre-yield toughness (Ty = 13.1 kN.mm) but a very high post-yield toughness (Tp = 653.18 kN.mm). The low Ty is consistent with its lack of a sharp elastic peak and early micro-cracking. The high Tp quantifies the “deflection-hardening” behavior and stable plateau. This energy is absorbed through the formation of a diffuse micro-crack zone at mid-span, where CNTs bridge nano-cracks, preventing localization and enabling sustained load resistance over large deformations (~ 21.8 mm).

The pre-yield (Ty), post-yield (Tp), and total toughness (Tt) values of all specimens are summarized in Table 6.

**Table 6:** Toughness values for all specimens.

Specimens	Pre-yield toughness (Ty) KN.mm	Post-yield toughness (Tp) KN.mm	Total toughness (Tt) KN.mm
CB-F	32.06	344.8	376.86
PGF1	38.7	583.7	622.4
WF1	45.6	498.6	544.2
PGF2	27.26	1349.63	1376.89
WF2	33.22	1342.43	1375.65
CNT-F	13.1	653.18	666.28
CB	67.78	371.38	439.16

#### Material action on energy absorption pathways

*Wire Mesh*: Primarily enhances pre-yield toughness (Ty) and provides robust post-yield toughness (Tp). Its high stiffness increases elastic energy storage and controls cracking, leading to a predictable, high-strength failure with considerable ductility. This is evident in WF1 (high Ty, high Tp).

*Geotextile*: Specializes in maximizing post-yield toughness (Tp), often at the expense of initial stiffness. Its compliant nature allows for crack opening, engaging large deformation mechanisms (fiber tension, pull-out) that absorb immense energy, as quantifiably proven by GS1’s exceptional Tp. This is ideal for collapse resistance and warning.

*Carbon Nanotubes (CNTs)*: Uniquely produce high post-yield toughness (Tp) from a low pre-yield toughness (Ty) base. They modify the matrix itself, transforming the failure process from localized cracking to distributed micro-damage. This results in superior crack control, durability, and a non-catastrophic, energy-absorbing failure, as shown by CNF’s high Tp relative to its Ty.

### Correlation of crack mechanics with macroscopic results

The fundamental link between the microscopic crack patterns and the macroscopic load-deflection behavior is governed by the principles of fracture mechanics and composite action.

1. *From Brittle to Ductile*: Concrete is a quasi-brittle material. Its inherent weakness is crack localization. All successful reinforcements work by modifying the crack-tip process zone or providing bridging stresses.
  *CNTs* act *ahead* of the crack tip, increasing the energy required to initiate and propagate micro-cracks (increased fracture energy).
  *Wire Mesh & Geotextiles* act *behind* the crack tip, applying closing forces (σ_br_) that reduce the stress intensity factor at the crack tip, thereby arresting its propagation.

2. *The Load-Deflection Curve as a Crack Signature*:
  *Linear Elastic Phase*: Governed by uncracked concrete. Slope indicates stiffness.
  *Curve Deviation/First Peak*: Marks the initiation of the first major macro-crack. A sharp peak indicates unstable propagation (CF). A rounded peak or plateau indicates stable, controlled cracking (GF1, CNF).
  *Post-Peak Plateau*: Represents the steady-state cracking phase. The plateau’s length equals the reinforcement’s ability to allow stable crack opening. Its height equals the residual bridging capacity of the reinforcement.
  *Final Drop*: Signifies the failure of the bridging mechanism (fiber/mesh rupture, debonding, or pull-out).

3. *Energy Absorption (Toughness)*: The area under the curve is the total energy dissipated. This energy is consumed by:
  Creating new crack surfaces (surface energy).
  Frictional pull-out of fibers/mesh from the matrix.
  Plastic deformation of the reinforcement. Beams with multiple cracks or large bridged cracks (GF1, GS1) have vastly larger energy absorption due to these combined mechanisms.

### Theoretical analysis of flexural capacity

Nominal flexural capacity (Mn) was calculated per ACI 318‑14^33^, with additional tension from geotextile or wire mesh added for internal specimens. For external specimens, ACI 440.2R‑17^34^ was adapted with bond reduction factor κ_m_ = 0.85. The woven geotextile modulus of 5.2 GPa and tensile strength of 45 kN/m were used. The experimental and theoretical ultimate load (Pu) results are summarized in Table 7.

**Table 7:** Experimental vs. theoretical ultimate load (Pu).

Specimen	Pu_exp_ (kN)	Pu_theo_ (kN)	Ratio (exp/theo)
RB-F	38.5	36.2	1.06
PGF1	55.1	52.3	1.05
WF1	56.2	58.1	0.97
PGF2	53.9	49.8	1.08
WF2	46.0	50.2	0.92
CNT-F	36.8	39.1	0.94

The average exp/theo ratio is 1.00 (COV = 6.2%), validating the experimental methodology.

### Synthesis: comparative assessment of strengthening mechanisms

Mechanism A: Distributed Internal Reinforcement – The woven geotextile (PGF1) provides high tensile strength (45 kN/m) and moderate elongation (12%), yielding a stable post‑cracking plateau. PGF1 reaches 55.1 kN (+ 43%). WF1 achieves the highest strength (56.2 kN, + 45%).

Mechanism B: External U‑Wrap – Woven geotextile externally wrapped (PGF2) achieves extreme ductility (µ = 20.1) due to inter‑yarn sliding and gradual debonding, with toughness 1376.9 kN·mm.

Mechanism C: Matrix Modification (CNT‑F) – CNTs do not increase peak strength but alter failure to pseudo‑ductile (µ = 11.5, Tp = 653.2 kN·mm).

Design Recommendations: Strength‑critical → WF1; energy absorption/seismic → PGF2; durability/crack control → CNT‑F.

### Comparative analysis with existing literature

The flexural performance enhancements observed in this study are compared with previously reported results for similar strengthening systems.

Wire Mesh Strengthening: Chandramouli et al.^25^ reported a 28% increase in ultimate load for internally welded wire mesh reinforced beams, compared to 45% increase for WF1 in the present study. The difference is attributed to the higher mesh density (50 × 50 mm vs. larger spacing) and superior bond characteristics achieved through the U-wrapping configuration. Mebrahtom et al.^24^ observed ductility indices of 4.2–5.8 for externally wrapped wire mesh, whereas WF2 achieved µ = 16.3, demonstrating the enhanced deformation capacity from the epoxy-bonded U-wrap system.

Geotextile Strengthening: Siddika et al.^18^ reported a 34% increase in flexural capacity and 112% increase in toughness for geotextile-wrapped beams. The present study achieved 43% strength increase (PGF1) and 266% toughness increase (PGF2, Tt = 1376.9 kN·mm vs. 376.9 kN·mm for control), demonstrating superior performance due to the combination of CNT-modified matrix and optimized U-wrap geometry.

CNT Modification: El-Feky et al.^26,27^ reported toughness enhancements of 150–200% for CNT-modified beams, consistent with the 77% increase in total toughness observed for CNT-F (666.3 kN·mm vs. 376.9 kN·mm for control). The pseudo-ductile failure mode (µ = 11.5) aligns with observations by Salman et al.^28^, who reported ductility indices of 8–12 for CNT-reinforced specimens.

## Conclusion

This experimental study investigated the flexural performance of RC beams strengthened with eco-friendly materials (woven geotextile and steel wire mesh) as internal or external reinforcement, combined with CNT matrix modification. Based on the experimental results, the following conclusions are drawn:

*Strength Enhancement*:

- Internal wire mesh (WF1) achieved the highest ultimate load of 56.2 kN, representing a 45% increase over the control beam (38.5 kN). Internal woven geotextile (PGF1) showed comparable performance with 55.1 kN (+ 43%).
- External strengthening provided moderate strength gains (PGF2: 53.9 kN, + 40%; WF2: 46.0 kN, + 19%) but delivered superior ductility.
- CNT-F did not increase peak strength (36.8 kN, -4% relative to control) but fundamentally altered the failure mechanism to pseudo-ductile behavior.

*Ductility and Energy Absorption*:

- External woven geotextile (PGF2) exhibited exceptional ductility (µ = 20.1) and total toughness (Tt = 1376.9 kN·mm), representing a 3.6-fold increase over the control beam. This makes PGF2 particularly suitable for seismic applications where energy dissipation is critical.
- CNT-F achieved µ = 11.5 and post-yield toughness Tp = 653.2 kN·mm through distributed micro-cracking, offering superior crack control for durability-critical applications (e.g., bridge decks, marine structures).

*Failure Mechanisms*:

- Internal reinforcement (WF1, PGF1) failed through reinforcement yielding/rupture after multiple cracking, providing predictable strength-based design.
- External reinforcement (WF2, PGF2) failed through progressive debonding, enabling deflections exceeding 30 mm and providing ample warning before collapse.
- CNTs promoted crack deflection and branching, resulting in diffuse micro-damage without localized macro-crack formation.

*Practical Recommendations for Real-World Applications*:

- *Strength-critical applications* (e.g., heavy industrial floors, bridge girders): WF1 (internal wire mesh) provides the highest load capacity with good ductility.
- *Seismic retrofitting and energy absorption* (e.g., earthquake-prone regions, impact-resistant structures): PGF2 (external geotextile U-wrap) offers exceptional ductility and toughness with minimal strength compromise.
- *Durability and crack control* (e.g., water-retaining structures, marine environments, tunnels): CNT-F provides distributed micro-cracking and enhanced fracture toughness without increasing section size.

*Contribution to Existing Knowledge*:

This study provides the first systematic comparison of internal versus external placement for woven geotextiles and wire mesh in flexural strengthening, and demonstrates the synergistic potential of combining CNT matrix modification with discrete macro-reinforcement. The results establish quantitative benchmarks for sustainable strengthening alternatives to conventional FRP systems.

*Limitations and Future Research*:

- *Limitations*: Small-scale specimens (600 × 150 × 150 mm), monotonic loading only, single U-wrap configuration, ambient curing conditions.
- *Future work*: Full-scale beam validation (span ≥ 3000 mm), cyclic/reverse cyclic loading for seismic assessment, long-term durability under freeze-thaw and wet-dry cycles, hybrid systems combining CNT matrix with external wraps, life-cycle cost and environmental impact assessment.

## Data Availability

All data generated or analyzed during this study are included in this published article.
